# Evodiamine Inhibits Gastric Cancer Cell Proliferation *via* PTEN-Mediated EGF/PI3K Signaling Pathway

**DOI:** 10.1155/2021/5570831

**Published:** 2021-11-16

**Authors:** Ruichuang Yang, Jianxia Wen, Tao Yang, Chunmei Dai, Yanling Zhao

**Affiliations:** ^1^College of Pharmacy, Jinzhou Medical University, Jinzhou 121000, China; ^2^Department of Pharmacy, Chinese PLA General Hospital, Beijing 100039, China

## Abstract

**Aims:**

In this study, the pharmacological effects and potential molecular mechanisms of evodiamine in treating gastric cancer (GC) were investigated.

**Methods:**

GC cells lines of AGS and BGC-823 were treated with evodiamine at various concentrations for different times (24, 48, and 72 h). Inhibition of the proliferation of AGS and BGC-823 cells was assessed using a CCK-8 assay. The morphology of gastric cancer cells was detected by high-content screening (HCS). The apoptosis-inducing effect of evodiamine on AGS and BGC-823 cells was detected by flow cytometric analysis. Cell migration and invasion were detected by Transwell assay. The relative mRNA and protein expression levels of PTEN-mediated EGF/PI3K signaling pathways were investigated *via* RT-qPCR or western blotting, respectively.

**Results:**

Evodiamine substantially inhibited AGS and BGC-823 cells proliferation in a dose- and time-dependent manner. Flow cytometric analysis revealed that evodiamine could induce apoptosis of AGS and BGC-823 cells in a dose-dependent manner. In addition, evodiamine inhibited AGS and BGC-823 cell migration and invasion. Mechanistically, the results demonstrated that evodiamine promoted the relative mRNA and protein expression of PTEN and decreased expression of EGF, EGFR, PI3K, AKT, p-AKT, and mTOR. Most importantly, evodiamine could effectively increase the mRNA and protein expression of PTEN and decrease the protein expression of EGF/PI3K pathway, indicating that evodiamine downregulated EGF/PI3K through the activation of PTEN pathway.

**Conclusion:**

Evodiamine inhibited the directional migration and invasion of GC cells by inhibiting PTEN-mediated EGF/PI3K signaling pathway. These findings revealed that evodiamine might serve as a potential candidate for the treatment or prevention of GC.

## 1. Introduction

Gastric cancer (GC) is a malignant tumor originating from gastric mucosal epithelium. It is the fifth most common cancer as well as the third most lethal of cancer death worldwide [1]. Eastern Asian countries account for approximately half of gastric carcinoma globally. In view of the high incidence rate, gastric cancer is a major burden of society [2]. In China, the incidence and mortality rate of gastric carcinoma are far higher than the world average level, coupled with complex etiology, low survival rate, and poor prognosis, which has become the main factor restricting the diagnosis and treatment of gastric cancer [3]. Therefore, there is a huge demand for promising agents and new therapies for the prevention and treatment of gastric cancer.

Plant-derived compounds are considerable source of anticancer drugs. In recent decades, the number of anticancer compounds has been extracted from natural sources [4]. Natural compounds and their derivatives isolated from Chinese herbal medicines are considered to be potential anticancer drugs and new adjuvants to improve the clinical efficacy of chemotherapeutic agents [5]. Evodiamine is a natural beta-carboline alkaloid extracted from the fruit of *Euodia rutaecarpa* (*Wu-Zhu-Yu* in Chinese). Traditionally, evodiamine has been commonly traditionally used to cure headache, amenorrhea, postpartum hemorrhage, and gastrointestinal diseases [6]. The multiple bioactive properties of evodiamine have been widely investigated, including antitumor [7], anti-inflammatory [8], antibacterial [9], neuroprotective [10], vasodilation [11], and antigastrointestinal motility [12]. Among them, the multitargeting molecule effect of evodiamine on the gastrointestinal is attractive. Studies have found that evodiamine has inhibitory effects on SGC-7901 cells, which is associated with apoptosis, autophagy, and cell cycle arrest at the G2/M phase in a dose-dependent manner [13]. Moreover, the potential mechanism is involving the downregulation of survivin and upregulation of caspase-3, -8, and -9 and altering the expression of caspase-3, Bax, and Bcl-2 [14, 15]. In addition, evodiamine inhibited the Wnt/*β*-catenin signaling pathway to inhibit proliferation and stem cell properties of GCSCs and repressed the EMT [16]. The present findings suggest that evodiamine is an effective natural compound for the treatment of gastric cancer. However, the antitumor effect and potential mechanism of evodiamine on gastric cancer cells remain to be further elucidated.

Phosphatase and tensin homolog (PTEN), a tumor-suppressor gene located on chromosome 10, is one of the key factors in the diagnosis of gastric cancer prognosis [17, 18]. Studies have shown that PTEN gene inactivation is closely related to cell apoptosis, cell proliferation, cell migration, and cell metastasis, resulting in association with the progression and incidence rate of gastric cancer [19, 20]. Conversely, patients with lower expression of PTEN protein have high probability in distant metastasis and advanced clinical stage of gastric cancer than in adjacent nontumor tissues [21]. The upregulation of epidermal growth factor (EGF), its receptor (EGFR), and ErbB2 protein in gastric mucosa plays an important role in the occurrence and development of gastric cancer [22]. EGF stimulates cell proliferation and migration by interacting with receptors and triggers epithelial cell signaling [23].

Phosphatidylinositol-3-kinase (PI3K) signaling pathway plays an important role in various aspects in terms of cell growth and cell survival by regulating cell cycle, differentiation, transcription, and apoptosis [24]. The imbalance of PI3K class I signaling pathway, whether through gene amplification or mutation, is closely related to the occurrence and development of a variety of cancers [25]. The genetic changes of proteins in this signaling pathway include PTEN and Akt [26]. The imbalance of PI3K pathway can induce a variety of downstream effectors, including mammalian target of rapamycin (mTOR). mTOR is a member of the PI3K-related kinase (PIKK) family. Its catalytic kinase domain is highly homologous to that of PI3K [27]. PI3K/mTOR pathway is one of the most common activated signaling pathways in human tumors [28]. Previous study indicated that evodiamine-induced PC cell apoptosis by inhibiting PI3K/AKT and mitogen-activated protein kinase/ERK and inhibiting the phosphorylation of signal transducer and activator of transcription activator 3 in human pancreatic cancer cells to inhibit autophagy [29], suggesting that evodiamine may be considered as a novel pancreatic cancer treatment. However, whether evodiamine plays an antigastric cancer effect by regulating EGF/PI3K signaling pathways remains to be further investigated. Thus, this study aimed to explore the effect of evodiamine on EGF/PI3K signaling pathways, which contributed to better understanding of the anticancer mechanisms of evodiamine.

## 2. Materials and Methods

### 2.1. Drugs and Chemicals

Standards of evodiamine (purity ≥ 98%, Cat no. CHB190217, CAS no. 518-17-2) was obtained from Chengdu Chroma Biotechnology Co., Ltd. (Chengdu, China). All drugs were dissolved in pure dimethyl sulfoxide (DMSO) and then diluted to indicated concentrations when used for AGS and BGC-823 cells.

### 2.2. Cell Culture

Human AGS and BGC-823 cell lines were obtained from the Cell Resource Centre (IBMS, CAMS/PUMC, Beijing, China). Cells were cultured in RPMI-1640 medium supplement 10% fetal bovine serum (FBS), containing 100 IU/mL penicillin and 100 *μ*g/mL streptomycin. Cells were incubated at normal culture conditions (37°C in a saturated humidity atmosphere with 95% air and 5% CO_2_).

### 2.3. Cell Viability Assay

Cell viability and proliferation was measured using the cell counting kit-8 (CCK-8, Cat. no. HY-KO301, MedChemExpress, USA) in line with the manufacturer's instructions. Briefly, AGS and BGC-823 cells were placed in 96-well plates. Then, cells were respectively treated with evodiamine at various concentrations (1.5625, 3.125, 6.25, 12.5, 25, 50, 100, and 200 *μ*M) for different times (24, 48, and 72 *μ*M). After drug intervention, 10 *μ*L of CCK-8 solution was added to each well and incubated at 37°C for 30 min. The absorbance was measured at 450 nm using a Synergy™ H1 instrument (BioTek, American). The cytotoxicity of evodiamine was measured, and rate of cell growth inhibition was calculated in accordance with the instructions. Each experiment was performed at least three times independently under each corresponding experimental condition.

### 2.4. Flow Cytometric Analysis of Cell Apoptosis

Human AGS and BGC-823 cells were treated with 6.25, 12.5, and 25 *μ*M evodiamine for 24 h. Next, the cells were collected, washed, and resuspended in PBS. The apoptotic cells were counted by Annexin V-FITC and 7-amino-actinomycin (Apoptosis detection kit; BD Biosciences, San Jose, CA, USA) double staining using the Annexin V-FITC Apoptosis Detection kit according to the manufacturer's instructions. The early apoptotic cells, late apoptotic cells, and necrotic cell death were detected by Annexin V positive, Annexin V and 7-AAD positive, and 7-AAD positive, respectively. The cells were washed twice with cold PBS and then resuspended in annexin-V-binding buffer at a concentration of 1 × 10^6^ cells/mL. Transfer 100 *μ*L of the solution to a 5 mL culture tube. The suspension was stained with 5 *μ*L of annexin V-FITC and 5 *μ*L of 7-AAD and incubated for 15 min at room temperature in the dark condition. Then, 400 *μ*L of 1 × binding buffer was added to each tube. Cell apoptosis rate was detected using a cytofluorimeter and analyzed by FACScan and CellQuest software (Becton, Dickinson).

### 2.5. High-Content Analysis

The nuclear morphology and cell proliferation of gastric cancer cells were detected by a High-Content System (Thermo Scientific, MA, USA). The localization, cell morphology, and quantitative analysis of AGS and BGC-823 cells were investigated by three fluorescent dyes, including Hoechst 33342 (H3570, Invitrogen), Calcein AM (C3099, Invitrogen), and EthD-1 (L3224, Invitrogen). In HCS system, cell health profiling assay module was chosen and corresponding wavelength channels were set to catch fluorescence images. The parameters and formats of the measurements were similar to our previous study [30]. Finally, an Array Scan XTI was performed to quantify the mean fluorescence intensity of gastric cancer cells.

### 2.6. Wound-Healing Assay

Wound-healing assay is performed to measure cell migration and repair ability. AGS and BGC-823 cells were seeded into 6-well plates. Cells were scraped off with the end of a 10 *μ*L pipette tip while grew to a certain density. Then, the detached cells were removed by washing the plate with PBS; the remaining cells were cultured in the medium containing 0, 12.5, and 25 *μ*M evodiamine solution for another 24 h. Cell migration and repair ability was detected under a 40x magnification phase contrast microscope after 0 and 24 h. AGS and BGC-823 cells migrated to the scratch area in 6 random fields were quantitatively analyzed by computer-assisted microscope.

### 2.7. Transwell Migration Assay

The migration ability of AGS and BGC-823 cells were evaluated by Transwell assay, which examined the ability of the cells to move under the membrane filter. Cell migration ability was conducted using Transwell polyester membrane filter inserts with 8 *μ*m pores (Corning Inc., Corning, NY, USA). Cells were treated with 0, 12.5, and 25 *μ*M evodiamine solution for 24 h. Cells on the top of the Transwell chambers were removed, and the migrated or invasion cells on the bottoms of the membranes were fixed with 4% paraformaldehyde, followed by staining with 0.1% crystal violet (Beyotime, Haimen, China). Migratory cells were imaged and counted under a light microscope (Olympus, Tokyo, Japan). Finally, three random fields of view for each transmembrane were analyzed and averaged.

### 2.8. Real-Time PCR

Total RNA was extracted from AGS and BGC-823 cell using TRIzol reagent (Nordic Bioscience, Beijing, China) and reversed into cDNA by transcription kit (Promega, Madison, USA). Relative gene expression levels of PTEN, EGF, EGFR, ErbB2, PI3K, AKT, mTOR, and GAPDH were detected by quantitative real-time PCR using cDNA and SYBR Green PCR Master Mix (Nordic Bioscience, Beijing, China). RT-PCR was performed on a QuantStudio™ Real-Time PCR System version 1.3 (Applied Biosystems by Thermo Fisher Scientific). Data were calculated as average fold changes compared to control group after normalization to *β*-actin. The primer sequences for real-time PCR analyses of mRNA expression in this study are listed in Table 1.

### 2.9. Western Blotting

Cells were lysed with RIPA buffer (Lot. no. 20190711, Solarbio, Beijing, China) in the presence of PMSF (Lot. no. 20190929, Solarbio, Beijing, China) at 4°C. After incubation for 15 min, the lysates were centrifuged at 15,000 ×g for 10 min at 4°C. The protein concentration of the lysates was detected with BCA protein assay kit (Lot. no. 20200319, Solarbio, Beijing, China) in line with the manufacturer's instructions. After boiling at 95°C for 5 min, the soluble lysates were completely mixed with 4 × SDS sample buffer (Lot. no. 20201025, Solarbio, Beijing, China). An equal amount of protein was isolated by SDS-polyacrylamide gel electrophoresis (SDS-PAGE) followed by Western blotting. The related protein was transferred to polyvinylidene fluoride (PVDF) membranes and the primary antibody, PTEN polyclonal antibody (Catalog number: 22034-1-AP, Proteintech, USA, dilution: 1 : 2,000), EGF polyclonal antibody (Catalog number: 27141-1-AP, Proteintech, USA, dilution: 1 : 500), EGFR (C-Terminal) polyclonal antibody (Catalog number: 51071-2-AP, Proteintech, USA, dilution: 1 : 1,000), p-PI3K (Catalog number: ab182651, Abcam, UK, dilution: 1 : 500), PI3K (Catalog number: 67121-1-Ig, Proteintech, USA, dilution: 1 : 2,000), AKT polyclonal antibody (Catalog number: 10176-2-AP, Proteintech, USA, dilution: 1 : 2,000), p-AKT (Catalog number: 66444-1-Ig, Proteintech, USA, dilution: 1 : 1,000), mTOR monoclonal antibody (Catalog number: 66888-1-Ig, Proteintech, USA, dilution: 1 : 5, 000), Beclin-1 (Catalog number: 66665-1-Ig, Proteintech, USA, dilution: 1 : 1,000), Bcl-2 (Catalog number: 3498S, Cell Signaling Technology, USA, dilution: 1 : 1,000), Bax antibody (Catalog number: 2772S, Cell Signaling Technology, USA, dilution: 1 : 1,000), Caspase-3 antibody (Catalog number: 9662S, Cell Signaling Technology, USA, dilution: 1 : 1,000), Caspase-8 (1C12) Mouse mAb (Catalog number: 9746S, Cell Signaling Technology, USA, dilution: 1 : 1,000), and anti-beta actin (Catalog number: ab8226, Abcam, UK, dilution: 1 : 1,000) were detected. PVDF membranes were incubated with goat anti-rabbit IgG (H + L)/HRP antibody (bs-40295G-HRP, Bioss, Beijing, China, 1 : 20,000) for 1 h at room temperature. The antibody was detected by enhanced chemiluminescence. Data were normalized using *β*-actin as an endogenous control.

### 2.10. Data and Statistical Analysis

Data are presented as means ± standard deviation (SD) from at least three separate experiments. The IC_50_ curves were estimated by plotting percentage of viability from the triplicate treatment versus concentration. The IC_50_ value was defined and calculated using the 4-full-parameter equation as defined by the GraFit software version 5.0.4 from Erithacus Software (Surrey, UK). Statistical differences of the data were performed using an unpaired Student's *t*-test and ANOVA. GraphPad Prism Software (version 8.2.0) was used for data analysis. Statistical significance was defined as *P* < 0.05.

## 3. Results

### 3.1. Evodiamine Dose- and Time-Dependently Inhibited AGS and BGC-823 Cell Proliferation *In Vitro*

**Figure 1:**
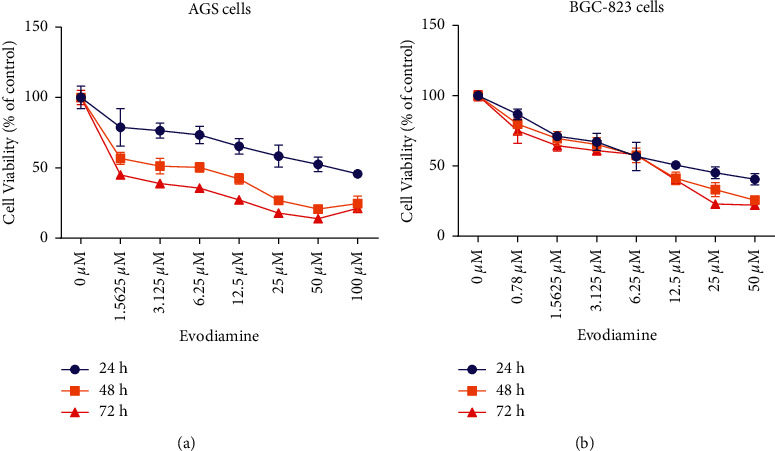
Effects of different concentrations and time points of evodiamine on human AGS and BGC-823 cell cytotoxicity. AGS cells were treated with 1.5625, 3.125, 6.25, 12.5, 25, 50, and 100 *μ*M evodiamine and BGC-823 cells were treated with 0.78, 1.5625, 3.125, 6.25, 12.5, 25, and 50 *μ*M evodiamine for 24, 48, and 72 h. CCK-8 assay was used to detect cell cytotoxicity. (a) Effects of different concentrations and time points of evodiamine on AGS cell cytotoxicity. (b) Effects of different concentrations and time points of evodiamine on BGC-823 cell cytotoxicity. Data were analyzed using one-way ANOVA followed by LSD. All data are presented as the mean ± SD.

To investigate the effect of evodiamine on gastric cancer cells, CCK-8 assay was performed to evaluate whether evodiamine affected cell proliferation of AGS and BGC-823 cells. Among them, AGS were treated with 1.5625, 3.125, 6.25, 12.5, 25, 50, and 100 *μ*M evodiamine at different times (24, 48, and 72 h). BGC-823 cells were treated with 0.78, 1.5625, 3.125, 6.25, 12.5, 25, and 50 *μ*M evodiamine for 24, 48, and 72 h. As shown in Figure 1(a) and Figure 1(b), evodiamine decreases cell proliferation of AGS and BGC-823 cells in a time- and concentration-dependent manner, which indicated that following a promotion in the evodiamine concentrations and time, the antiproliferation effects of evodiamine on AGS and BGC-823 cells gradually and substantially increased (*P* < 0.01). The IC_50_ of evodiamine on AGS cells in 24 h, 48 h, and 72 h was 63.84 ± 7.64, 6.69 ± 1.11, and 0.86 ± 0.07, respectively. The IC_50_ of evodiamine on BGC-823 cells in 24 h, 48 h, and 72 h was 16.90 ± 2.04, 7.16 ± 1.34, and 6.44 ± 0.62, respectively. When the concentration of evodiamine is 25 *μ*M, the cell viability of AGS and BGC-823 cells could be reduced to about 60% of that of the control group. Thus, AGS cells and BGC-823 cells were incubated with 25 *μ*M evodiamine for 24 h in the subsequent experiments, unless otherwise specified. Although evodiamine could decrease the proliferation of AGS and BGC-823 cells, the potential mechanism of evodiamine-induced cytotoxicity or cell arrest remains unclear.

### 3.2. Evodiamine Suppresses Wound-Healing of AGS and BGC-823 Cells

To explore the antimetastasis potential of evodiamine on gastric cancer cells, wound-healing assay was performed to evaluate the ability of evodiamine on cell migration.

**Figure 2:**
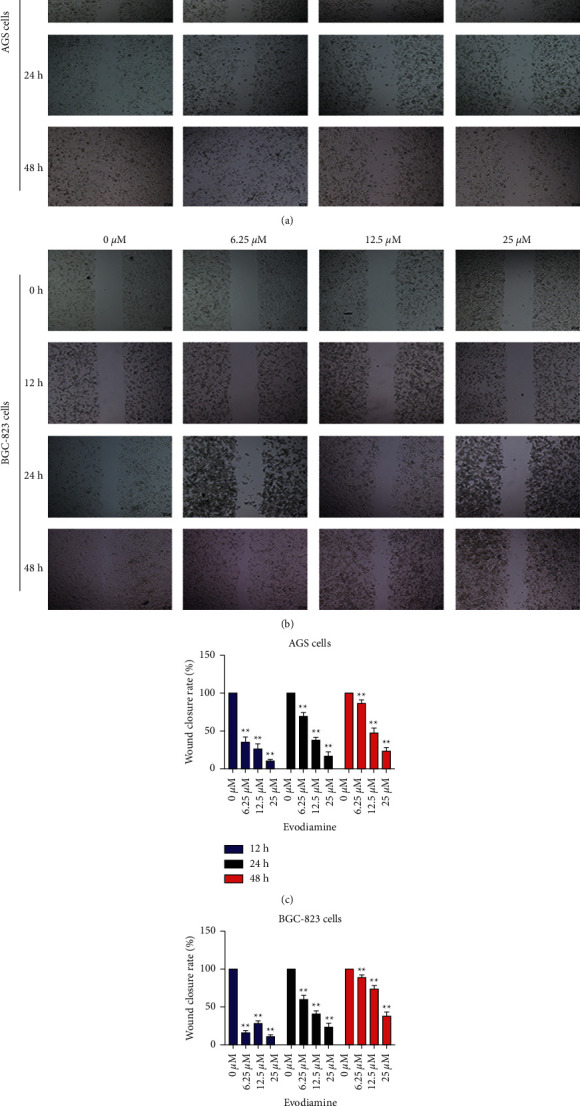
Evodiamine suppresses wound-healing of AGS and BGC-823 cells. Cells were treated with different concentration (6.25, 12.5, and 25 *μ*M) of evodiamine for 12, 24, and 48 h. Migration ability was observed using a wound-healing assay. (a) Representative photomicrographs for wound-healing assay of evodiamine on AGS cells. (b) Representative photomicrographs for wound-healing assay of evodiamine on BGC-823 cells. (c) Wound closure rate of AGS cells (% of control). (d) Wound closure rate of BGC-823 cells (% of control). ^∗∗^*P* < 0.01 versus control group (*n* = 3). Data were analyzed using one-way ANOVA followed by LSD. All data are presented as the mean ± SD.

At 0 h, a wound-healing was drawn between AGS and BGC-823 cells with a 10 *μ*L tip, and the effects of different concentrations of evodiamine on wound-healing were observed. The wound-healing migration assay indicated that the wound-healing ability of AGS cells and BGC-823 cells treated with different concentrations of evodiamine (6.25, 12.5, and 25 *μ*M) for 12, 24, and 48 h was significantly decreased in a dose-dependent manner relative to untreated controls (Figure 2). With the prolongation of time, the cells have different degrees of healing and migration tendency. After 12 h, compared with control group (0 *μ*M), a small number of AGS cells healed. After 24 hours, compared with the control group, each concentration of evodiamine had a certain effect on the wound-healing of AGS cells. 12.5 *μ*M and 25 *μ*M evodiamine groups could significantly inhibit AGS cell healing. AGS cells in 6.25 *μ*M groups almost completely healed at 48 h. However, the healing rate of 12.5 and 25 *μ*M groups decreased significantly with the increase of concentration. AGS cells in group 25 *μ*M did not completely heal even at 48 h (Figures 2(a) and 2(c)). As for BGC-823 cells, the wound closure rate of BGC-823 cells was low at 12 h. At 24 h, the wound-healing of BGC-823 cells was affected by different concentrations of evodiamine. BGC-823 cells in 6.25 *μ*M groups had healed, while in 12.5 *μ*M and 25 *μ*M groups were significantly inhibited. After 48 h, BGC-823 cells in 6.25 *μ*M group almost completely healed at 48 h. However, the wound closure rate of 12.5 *μ*M and 25 *μ*M groups decreased significantly with the increase of concentration (Figure 2(b)). BGC-823 cells in group 25 *μ*M did not completely heal even at 48 h (Figures 2(b) and 2(d)).

### 3.3. Evodiamine Suppresses AGS and BGC-823 Cell Migration

**Figure 3:**
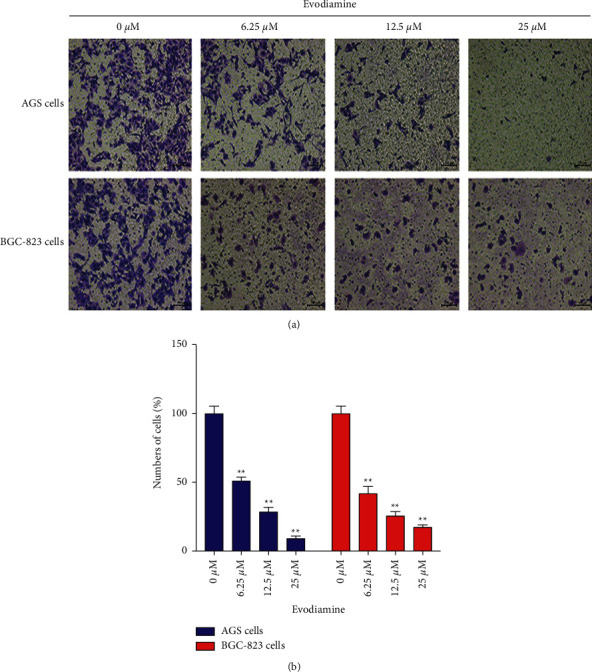
Evodiamine suppresses AGS and BGC-823 cell migration. Cells were treated with different concentration (6.25, 12.5, and 25 *μ*M) of evodiamine for 24 h. Migration ability was observed using a Transwell migration assay. (a) Representative photomicrographs for Transwell migration assay of evodiamine on AGS and BGC-823 cells. (b) Quantitative analysis of cell migration (% of control). ^∗∗^*P* < 0.01 versus control group (*n* = 3). Data were analyzed using one-way ANOVA followed by LSD. All data are presented as the mean ± SD.

To explore the antimetastasis potential of evodiamine on gastric cancer cells, cell invasion was performed to evaluate the ability of evodiamine on cell migration. The results showed that the invasion potential of AGS and BGC-823 cells in Transwell migration assay was effectively inhibited by evodiamine treatment (Figure 3(a)). Specifically, compared with the control group, 6.25 *μ*M, 12.5 *μ*M, and 25 *μ*M evodiamine groups could significantly reduce the migration rate of AGS and BGC-823 cells (*P* < 0.01), especially 12.5 *μ*M and 25 *μ*M groups (Figure 3(b)). The results showed that evodiamine significantly inhibited AGS and BGC-823 cell migration in a dose-dependent manner in the cell invasion assay (Figure 3). Therefore, both the wound-healing and cell invasion assays suggested that evodiamine could inhibit the motility of AGS and BGC-823 cells.

### 3.4. Evodiamine Inhibits Gastric Cancer Cell Proliferation

**Figure 4:**
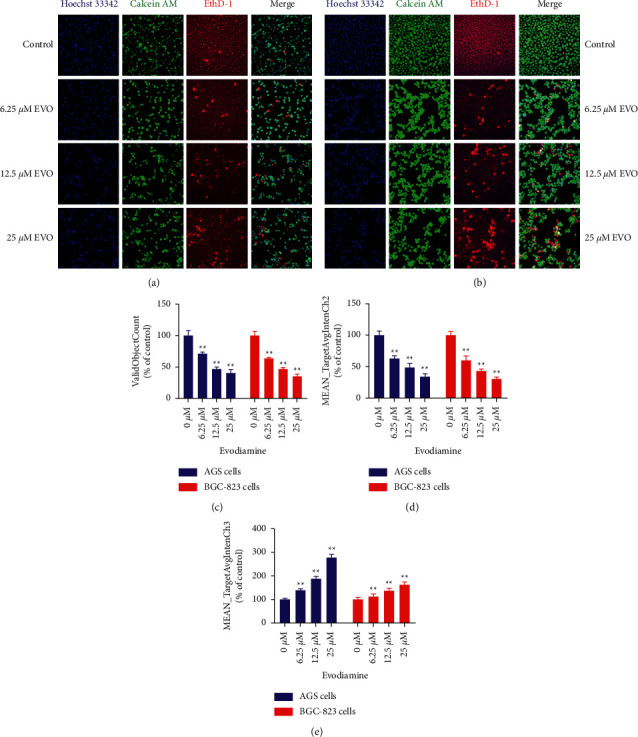
Evodiamine inhibits gastric cancer cell proliferation. Cells were treated with different concentrations (6.25, 12.5, and 25 *μ*M) of evodiamine for 24 h. Cell proliferation, viability, and morphology of AGS and BGC-823 cells were analyzed by HCA. (a) Representative photomicrographs for HCS image analysis of evodiamine on AGS and BGC-823 cells. (a) Cells count for HCS analysis of AGS and BGC-823 cells (% of control). (b) Live cells count of AGS and BGC-823 cells (MEAN_TargetAvgIntenCh2). (c) Dead cells count of AGS and BGC-823 cells (MEAN_TargetAvgIntenCh3). ^∗∗^*P* < 0.01 versus control group (*n* = 3). The results are expressed as percentages of control group. Data were analyzed using one-way ANOVA followed by LSD.

In order to intuitively display the effect of evodiamine on the morphology and proliferation of gastric cancer cells, cell proliferation, viability, and morphology of AGS and BGC-823 cells were analyzed by HCA. Cell nucleus (blue fluorescence), living cells (green fluorescence), and dead cells (red fluorescence) were labeled with Hoechst 33342, Calcein AM, and EthD-1, respectively. Representative photomicrographs for HCS image analysis of evodiamine on AGS and BGC-823 cells are shown in Figure 4(a). The results showed that blue fluorescence and green fluorescence were normal distribution in the nucleus and cytoplasm of control group. However, compared with the control group, AGS and BGC-823 cells were significantly decreased while treated with different concentrations (6.25, 12.5, and 25 *μ*M) of evodiamine. With the increase of concentration, the blue fluorescence and green fluorescence gradually decreased (Figures 4(c) and 4(d)), and the red fluorescence increased gradually (Figure 4(e)), indicating that the number of living cells decreased and the number of dead cells increased (Figure 4(e)). Evodiamine can significantly reduce the proliferation rate of gastric cancer cells, causing the nucleus fragmentation, cytoplasmic dissolution, and cell deformation of AGS (Figure 4(a)) and BGC-823 cells (Figure 4(b)). These results indicate that evodiamine could promote the proliferation of gastric cancer cells, destroy the normal morphology of gastric cancer cells, and produce cytotoxicity on gastric cancer cells.

### 3.5. Evodiamine Enhances Cell Apoptosis in Human Gastric Cancer Cells

**Figure 5:**
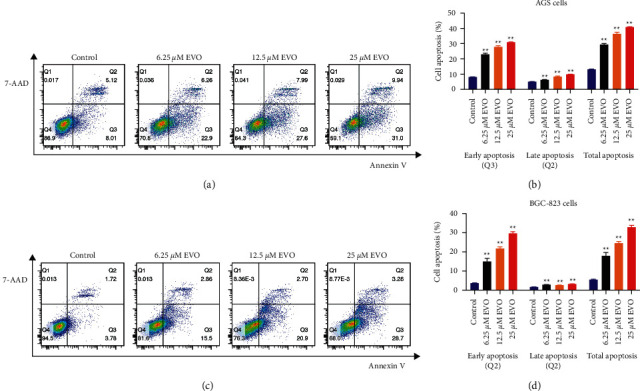
Evodiamine-induced early, late apoptosis of AGS and BGC-823 gastric cancer cells. Flow cytometric analysis accompanied with Annexin V-FITC/7-AAD. Double staining was used to investigate early, late, and total apoptosis in AGS and BGC-823 cells treated with various concentrations of evodiamine (6.25, 12.5, and 25 *μ*M). (a) Evodiamine-induced apoptosis of AGS gastric cancer cells. (b) Quantitative analysis of apoptotic cells in AGS gastric cancer cells. (c) Evodiamine-induced apoptosis of BGC-823 gastric cancer cells. (d) Quantitative analysis of apoptotic cells in BGC-823 gastric cancer cells. ^∗∗^*P* < 0.01 versus control group (*n* = 3). The results are expressed as percentages of control group. Data were analyzed using one-way ANOVA followed by LSD.

The apoptosis of AGS and BGC-823 cells was detected by flow cytometry following 24 h of evodiamine incubation. Compared with the control group, AGS and BGC-823 cells in different concentrations (6.25, 12.5, and 25 *μ*M) of evodiamine showed different degrees of apoptosis (Figures 5(a) and 5(d)). Quantitative analysis of apoptotic cells demonstrated that evodiamine significantly increased the rate of early apoptosis, late apoptosis, and total apoptosis in AGS (Figure 5(b)) and BGC-823 (Figure 5(d)) cells with the increase of evodiamine concentration (*P* < 0.01). The inhibitory effect of 25 *μ*M evodiamine showed the most significant effects, as determined by Annexin V-FITC/7-AAD double staining.

### 3.6. Effects of Evodiamine on the Relative mRNA Expression of PTEN-Mediated EGF/PI3K Signaling Pathways

**Figure 6:**
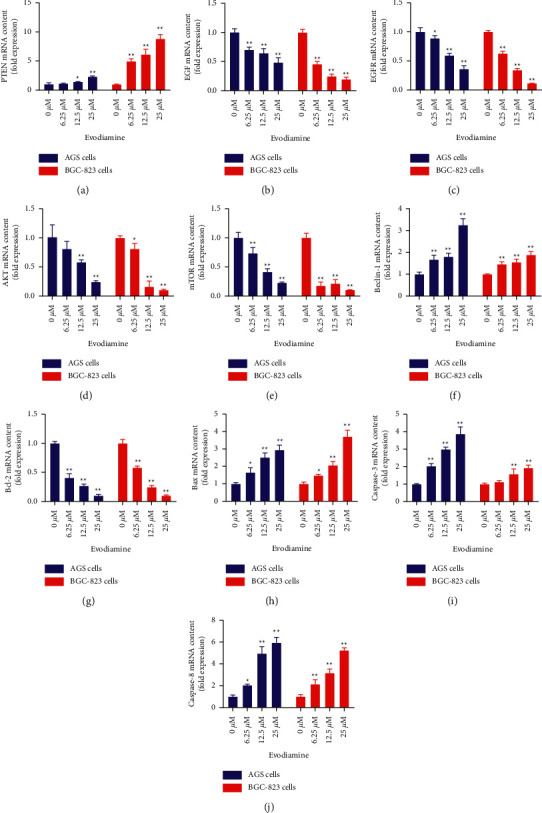
Effect of evodiamine on the gene expression level of PTEN-mediated EGF/PI3K signaling pathways. The relative mRNA expression levels of PTEN (a), EGF (b), EGFR (c), AKT (d), mTOR (e), Beclin-1 (f), Bcl-2 (g), Bax (h), caspase-3 (i), and caspase-8 (j) were detected by RT-PCR in different groups. ^*∗*^*P* < 0.05 and ^∗∗^*P* < 0.01 versus control group (0 *μ*M). Data were analyzed using one-way ANOVA followed by LSD. All data are presented as the mean ± SD (*n* = 3).

PTEN expression levels may be an important molecular issue in the occurrence and development of gastric cancer. Also, it can be regarded as a molecular marker as well as a reliable prognostic indicator of gastric cancer [31, 32]. To investigate the potential mechanisms by which evodiamine inhibits the relative mRNA expression of PTEN-mediated EGF/PI3K signaling pathways, AGS and BGC-823 cells were treated with different concentration of evodiamine (6.25, 12.5, and 25 *μ*M) for 24 h. Next, the mRNA expression levels of PTEN-mediated EGF/PI3K pathway (i.e., PTEN, EGF, EGFR, AKT, mTOR, Beclin-1, Bcl-2, Bax, caspase-3, and caspase-8) were measured by RT-PCR. As shown in Figure 6, evodiamine could significantly increase the relative mRNA expression of PTEN, Beclin-1, Bax, caspase-3, and caspase-8 (*P* < 0.01), while dramatically decrease the relative mRNA expression of EGF, EGFR, AKT, mTOR, and Bcl-2 (*P* < 0.01). The results indicated that evodiamine dramatically increased the expression levels of all apoptosis and autophagy-related genes to values found in AGS and BGC-823 cells. Thus, evodiamine could induce apoptosis of gastric cancer cells, indicating that it might have the potential to treat gastric cancer.

### 3.7. Effects of Evodiamine on the Relative Protein Expression of PTEN-Mediated EGF/PI3K Signaling Pathways

**Figure 7:**
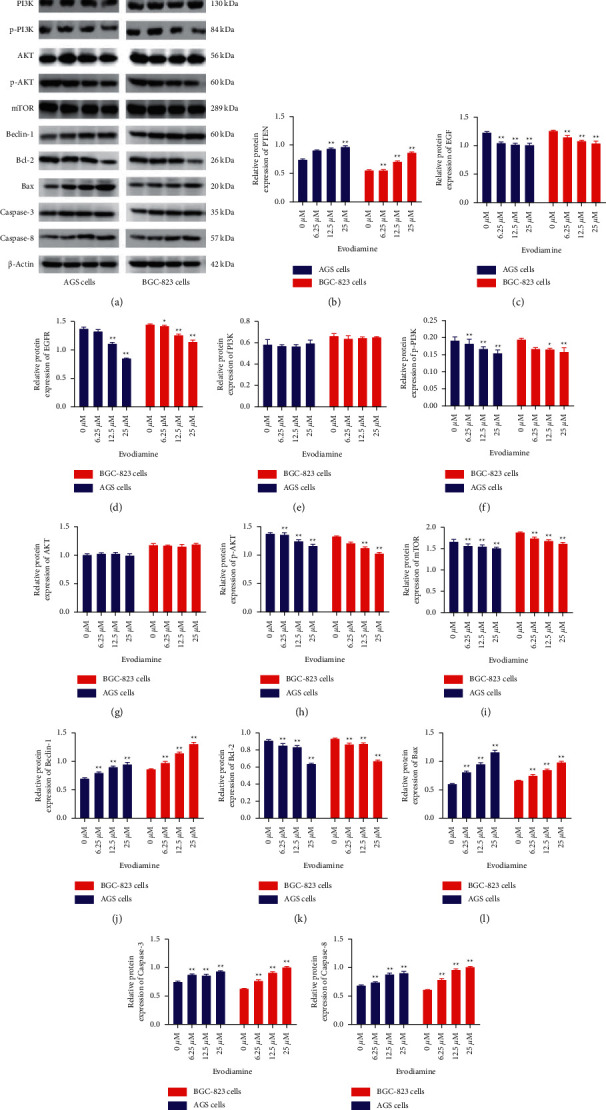
Effect of evodiamine on the protein expression level of PTEN-mediated EGF/PI3K signaling pathways. (a) Western blot images of PTEN-mediated EGF/PI3K signaling pathways. (b) Relative PTEN protein level in AGS and BGC-823 cells. (c) Relative EGF protein level in AGS and BGC-823 cells. (d) Relative EGFR protein level in AGS and BGC-823 cells. (e) Relative PI3K protein level in AGS and BGC-823 cells. (f) Relative p-PI3K protein level in AGS and BGC-823 cells. (g) Relative AKT protein level in AGS and BGC-823 cells. (h) Relative p-AKT protein level in AGS and BGC-823 cells. (i) Relative mTOR protein level in AGS and BGC-823 cells. (j) Relative Beclin-1 protein level in AGS and BGC-823 cells. (k) Relative Bcl-2 protein level in AGS and BGC-823 cells. (l) Relative Bax protein level in AGS and BGC-823 cells. (m) Relative caspase-3 protein level in AGS and BGC-823 cells. (n) Relative caspase-8 protein level in AGS and BGC-823 cells. ^*∗*^*P* < 0.05, ^∗∗^*P* < 0.01 versus control group (0 *μ*M). Data were analyzed using one-way ANOVA followed by LSD. All data are presented as the mean ± SD (*n* = 3).

To further confirm that PTEN plays an important role in cell apoptosis-related signaling pathway in gastric cancer cells, AGS and BGC-823 cells were treated with different concentrations of evodiamine (6.25, 12.5, and 25 *μ*M) for 24 h. Next, the protein expression levels of PTEN, EGF, EGFR, AKT, p-AKT, mTOR, Beclin-1, Bcl-2, Bax, caspase-3, and caspase-8 in different groups were detected (Figure 7(a)). Consistent with the expression of mRNA, compared to the control group, evodiamine treatment significantly increased the expression of PTEN, Beclin-1, Bax, caspase-3, and caspase-8 by the concentration-dependent way (*P* < 0.05, *P* < 0.01) (Figures 7(b), 7(h), and 7(j)–7(l)) but decreased EGF, EGFR, p-AKT, mTOR, and Bcl-2 both in AGS and BGC-823 cells (*P* < 0.05, *P* < 0.01) (Figures 7(c), 7(d), 7(f), 7(g), and 7(i)), indicating that evodiamine could promote the apoptosis of gastric cancer cells.

## 4. Discussion

The present study aimed to investigate the role of evodiamine, a natural active ingredient of the Traditional Chinese medicine *Evodia rutaecarpa*, in gastric cancer cells. The results revealed that evodiamine inhibited the proliferation of AGS and BGC-823 cells, destroying the structure and function of gastric cancer cells. The present study also provided the pharmacodynamics and mechanism evidence that the evodiamine has potential therapeutic effects of antigastric cancer in vitro. Based on the effects of promoting apoptosis and inhibiting migration of gastric cancer cells *in vitro*, evodiamine might serve as the active component of *Evodia rutaecarpa*. Furthermore, evodiamine inhibits gastric cancer cells' directional migration by inhibiting PTEN-mediated EGF/PI3K signaling pathways. This study explored the effect of evodiamine on the proliferation and migration of gastric cancer cells, indicating that evodiamine could inhibit the proliferation and metastasis of gastric cancer *in vitro*.

Numerous studies have shown that evodiamine has anticancer activity in various tumor cells and has inhibitory effect on gastric cancer [16], lung cancer [33], breast cancer cells [34], liver cancer [35], pancreatic cancer cells [36], ovarian cancer [37], chronic myeloid leukemia cells [38], and bladder cancer [5]. In this study, CCK-8 assay was used to assess the gastric cancer cells' viability *in vitro*. The results indicated that evodiamine could significantly inhibit AGS and BGC-823 cells' viability compared with the control groups. To directly display the effects of evodiamine on cell viability, HCS was used to qualitatively and quantitatively evaluate cell proliferation and morphology of gastric cancer cells. The results showed that evodiamine could significantly decrease the Hoechst and Calcein AM fluorescence intensity but substantially increase the EthD-1 fluorescence intensity of AGS and BGC-823 cells, indicating cell death compared with the control group. Notably, the nucleus and cytoplasm of AGS and BGC-823 cells were broken with incomplete morphology, indicating toxic effect of evodiamine on gastric cancer cells. It has been shown that inducing apoptosis is the key factor responsible for the anticancer role of evodiamine, which can alter the balance of relative gene and protein expression between proapoptotic Bax and antiapoptotic Bcl-2 family members and induce cell apoptosis by activating either effector caspase-3 or initiator caspase-8 [39]. In the present study, it was demonstrated that gastric cancer cells treated with evodiamine exhibited a concentration-dependent increase in the number of apoptotic and necrotic cells. Annexin V staining directly showed that it could induce apoptosis of gastric cancer cells. The results of qRT-PCR confirmed that 25 *μ*M evodiamine could upregulate the expression of proapoptotic Bax and downregulate the antiapoptotic Bcl-2 in gastric cancer cells, resulting in the upregulation of Bax/Bcl-2 ratio. In addition, it activates caspase-3, caspase-8, and caspase-9 genes in AGS and BGC cells, simultaneously. These results suggested that endogenous and exogenous apoptotic pathways are involved in evodiamine-induced apoptosis of gastric cancer cells.

Previous studies have reported a close association between apoptosis and tumor development [40, 41]. Therefore, the exploration of the mechanisms underlying drug-induced tumor cell apoptosis is essential for improving drug efficacy and the development of novel anticancer drugs. PTEN is one of the most common tumor-suppressor genes in cancer. PTEN deficiency showed overactivation of PI3K/AKT pathway activation markers and upregulation of mTOR [42]. In addition, previous study investigated the effects of evodiamine on human pancreatic cancer cell, and the results indicated that evodiamine-induced pancreatic cancer cell apoptosis by inhibiting PI3K/AKT and mitogen-activated protein kinase/ERK [29]. However, whether evodiamine regulates gastric cancer cell apoptosis through regulating PI3K/Akt and its upstream and downstream pathways has not been further explored. To clarify the potential action mechanisms of evodiamine, the mRNA and protein expression levels of PTEN-mediated EGF/PI3K signaling pathways were measured. As downregulation of PTEN decreases anticancer effects in various tumors, we speculated that the PTEN-mediated EGF/PI3K signaling pathways is responsible for the gastric cancer cell apoptosis and cell migration. Consistent with the previous report, the mRNA and protein expression levels of PTEN were markedly increased in the evodiamine treatment, but the levels of EGF, EGFR, ErbB2, PI3K, AKT, and mTOR were significantly decreased compared to the control group. Furthermore, the expression of PTEN was substantially decreased, while the AGS and BGC-823 cells were pretreated with VO-OHpic trihydrate. The mRNA and protein expression levels of EGF/PI3K signaling pathways increased in various degrees. However, evodiamine could inhibit this increase. These results suggest that evodiamine may play a role in the treatment of gastric cancer by regulating PTEN-mediated EGF/PI3K signaling pathways.

This study explored the anticancer effects and potential mechanism of evodiamine on gastric cancer cells *in vitro*. However, this study still has some limitations. Firstly, this study only examined the effects of evodiamine on the cell proliferation, apoptosis, and migration of AGS and BGC-823 gastric cancer cells. However, the effect of evodiamine on other gastric cancer cells, such as MKN-45, MKN-28, MGC-803, and HGC-27, needed further investigated. Secondly, this study only explored the anticancer effect of evodiamine *in vitro*, but not from the overall animal level and clinical patients *in vivo*, which led to the validation of conclusion being weaken. Lastly, there are many active compounds in *Euodia rutaecarpa*. Whether other compounds, such as rutaecarpine and dehydroevodiamine, have antigastric cancer activity remains to be further studied.

## 5. Conclusions

The results of the present study indicated that evodiamine-induced AGS and BGC-823 cell apoptosis may be associated with increased expression levels of PTEN and decreased expression levels of EGF/PI3K signaling pathway. Thus, evodiamine may present a potential and novel preventive candidate for gastric cancer *in vitro*. In addition, further studies are required to investigate the mechanism of other alkaloids in *Evodia rutaecarpa* for the treatment of gastric cancer *in vivo*.

## Figures and Tables

**Table 1:** Primer sequences for real-time PCR analyses of mRNA expression in this study.

Gene	Forward	Reverse
PTEN	GTGGTCTGCCAGCTAAAGGTGAAG	ACAGGTAACGGCTGAGGGAACTC
EGF	GTCTGCGTGGTGGTGCTTGTC	ACTCCTCACATCTCTGCTCGACTC
EGFR	GTGTGCCACCTGTGCCATCC	GTGTGCCACCTGTGCCATCC
Caspase-3	GTGGAGGCCGACTTCTTGTATGC	TGGCACAAAGCGACTGGATGAAC
Caspase-8	CGGATGAGGCTGACTTTCTGCTG	GGCTCTGGCAAAGTGACTGGATG
Bax	GATGCGTCCACCAAGAAGCTGAG	CACGGCGGCAATCATCCTCTG
Bcl-2	TACGAGTGGGATGCGGGAGATG	CCGGGCTGGGAGGAGAAGATG
Beclin-1	ACATCTGGCACAGTGGACAGTTTG	AGCATGGAGCAGCAACACAGTC
AKT	GCAGGATGTGGACCAACGTGAG	GCAGGCAGCGGATGATGAAGG
mTOR	CTTGCTGAACTGGAGGCTGATGG	CCGTTTTCTTATGGGCTGGCTCTC
*β*-actin	GGCCAACCGCGAGAAGATGAC	GGATAGCACAGCCTGGATAGCAAC

## Data Availability

The data used to support the findings of this study are available from the corresponding author upon reasonable request.

## Abbreviations

HCS: High-content screening
PTEN: Phosphatase and tensin homolog
EGF: Epidermal growth factor
PI3K: Phosphatidylinositol-3-kinase
DMSO: Dimethyl sulfoxide
PVDF: Polyvinylidene fluoride.
